# Pretravel Health Advice Among Australians Returning From Bali, Indonesia: A Randomized Controlled Trial Protocol

**DOI:** 10.2196/resprot.6549

**Published:** 2016-12-07

**Authors:** Chloe A Thomson, Robyn A Gibbs, Jane S Heyworth, Carolien Giele, Martin J Firth, Paul V Effler

**Affiliations:** ^1^ School of Population Health Faculty of Medicine and Dentistry The University of Western Australia Perth Australia; ^2^ Communicable Disease Control Directorate Department of Health Western Australian Government Perth Australia; ^3^ Centre for Applied Statistics School of Mathematics and Statistics The University of Western Australia Perth Australia; ^4^ School of Pathology and Laboratory Medicine Faculty of Medicine and Dentistry The University of Western Australia Perth Australia

**Keywords:** travel medicine, tropical medicine, travel, diarrhea, clinical trial, social media

## Abstract

**Background:**

The effect of pretravel health advice (PTHA) on travel-related illness rates is poorly understood, and to date there are no published randomized controlled trials evaluating the impact of PTHA outcomes.

**Objective:**

This study aims to determine the effect of an online PTHA intervention on travel-related illness rates in Western Australians visiting Bali, Indonesia.

**Methods:**

Western Australian travelers to Bali will be recruited online before departure and will be randomly allocated to an intervention or control group by computer algorithm. The intervention in this study is a short animated video, with accompanying text, containing PTHA relevant to Bali. An online posttravel survey will be administered to all participants within two weeks of their return from Bali. The primary outcome is the difference in self-reported travel-related illness rates between control and intervention groups. Secondary outcomes include the difference in risk prevention behaviors and health risk knowledge between the control and intervention groups. Further secondary outcomes include whether individuals in the control group who sought external PTHA differ from those who did not with respect to risk prevention behaviors, health risk knowledge, and health risk perception, as well as the rate of self-reported travel-related illness.

**Results:**

The study began recruitment in September 2016 and will conclude in September 2017. Data analysis will take place in late 2017, with results disseminated via peer-reviewed journals in early 2018.

**Conclusions:**

This will be the first randomized controlled trial to examine the effect of a novel PTHA intervention upon travel-related illness. In addition, this study builds upon the limited existing data on the effectiveness of PTHA on travel-related illness.

**ClinicalTrial:**

Australian New Zealand Clinical Trials Registry (ANZCTR): ACTRN12615001230549; https://www.anzctr.org.au/Trial/Registration/TrialReview.aspx?id=369567 (Archived by WebCite at http://www.webcitation.org/6m0G7xJg1)

## Introduction

### Australian Travel Overseas

Increasing numbers of Australians are traveling overseas. The number of short-term international departures from Australia doubled to over 9.4 million in 2015, compared to the previous 10 years [1].

Bali, Indonesia has been a popular holiday destination for Australians since the 1970s. Western Australian (WA) travelers now account for nearly half of the total Australian visitors to Indonesia, increasing more than six-fold between 2006 and 2015 [1]. There were over 454,000 departures from Perth to Bali in 2015, with nine flights, on average, every day [2]. Travelers from Perth to Bali are the focus of our study.

### Rates of Travel-Related Disease in Returning Travelers

Reported rates of travel-related illness vary, with studies estimating that 22-64% of travelers experience some form of health impairment while traveling, depending on the destination and season of travel [3]. In Western Australia, almost 8% of communicable disease notifications are for illnesses acquired overseas. From 2006 to 2015, the proportion of overseas-acquired infections in Western Australia attributed to travel to Indonesia rose from 10% to 42% (personal communication from Paul Saunders, Data Custodian for the Western Australian Notifiable Infectious Diseases Database at the Western Australian Department of Health, April 7, 2016) [4]. The most common notifiable diseases acquired in Indonesia are dengue fever and gastroenteritis caused by *Campylobacter* and *Salmonella* species [4].

Establishing an accurate picture of travel-related infections is difficult due to underdiagnosis and under-reporting, the highly transient nature of international travel, and the emergence of new disease risks in travel destinations. In particular, while notifiable disease data are available, there are no comparable data available for non-notifiable illnesses, such as traveler’s diarrhea, among residents returning to Western Australia.

### Pretravel Health Advice in Travelers

Delivering appropriate and effective pretravel health advice (PTHA) to travelers may be important to reduce the risk of illness to travelers and to prevent the importation of travel-related diseases [5]. The average proportion of travelers seeking professional PTHA globally, as reported in airport surveys, is approximately 48% [5]. Travelers from the United States, Australia, and Asia are reported to consistently fall below this average [6-9], with Canadian and European travelers reporting PTHA more frequently [10-12].

### Outcomes of Pretravel Health Advice: Rate of Illness

There are limited and conflicting data on the impact of PTHA in reducing travel-related illnesses. A retrospective cohort study in Scotland found that people who consulted a travel doctor prior to traveling overseas were less likely to become ill than those who had seen a general practitioner [13]. However, of the 1668 participants, only 100 had attended a travel clinic. An Italian retrospective study of 300 travelers to malaria-endemic countries showed that visiting a travel clinic pretravel was protective against travelers’ diarrhea, but not against fever [14]. In an analysis of European surveillance data, travelers falling ill abroad were less likely to present with malaria, acute hepatitis, HIV, or animal bites requiring postexposure prophylaxis if they had received PTHA [15].

Conversely, some researchers have concluded that PTHA does not protect against illness in travelers [16-19]. It is proposed that people who take advice prior to travel may already be more aware of travel illnesses, possibly due to previous exposure or personal susceptibility [16,17]. In a recent prospective cohort study of 1277 patients at a Swedish travel clinic, illness after travel was compared between those who complied with the advice and those reporting noncompliance or inattention to PTHA provided [19]. Self-reported compliance with PTHA was not found to be protective against illness while travelling.

The observational studies cited above demonstrate discordant results regarding the effect of PTHA on reducing travel-related illness. However, methodological issues such as selection bias and sample size limit the inferences that can be drawn from these studies. To address these issues, our proposed study incorporates a randomized controlled trial design on a large cohort to evaluate the effect PTHA has on reported illness. Specifically, the aim of the study is to determine the effect of an online PTHA intervention on travel-related illness rates and behaviors in WA travelers visiting Bali, Indonesia.

## Methods

### Study Design

**Figure 1:**
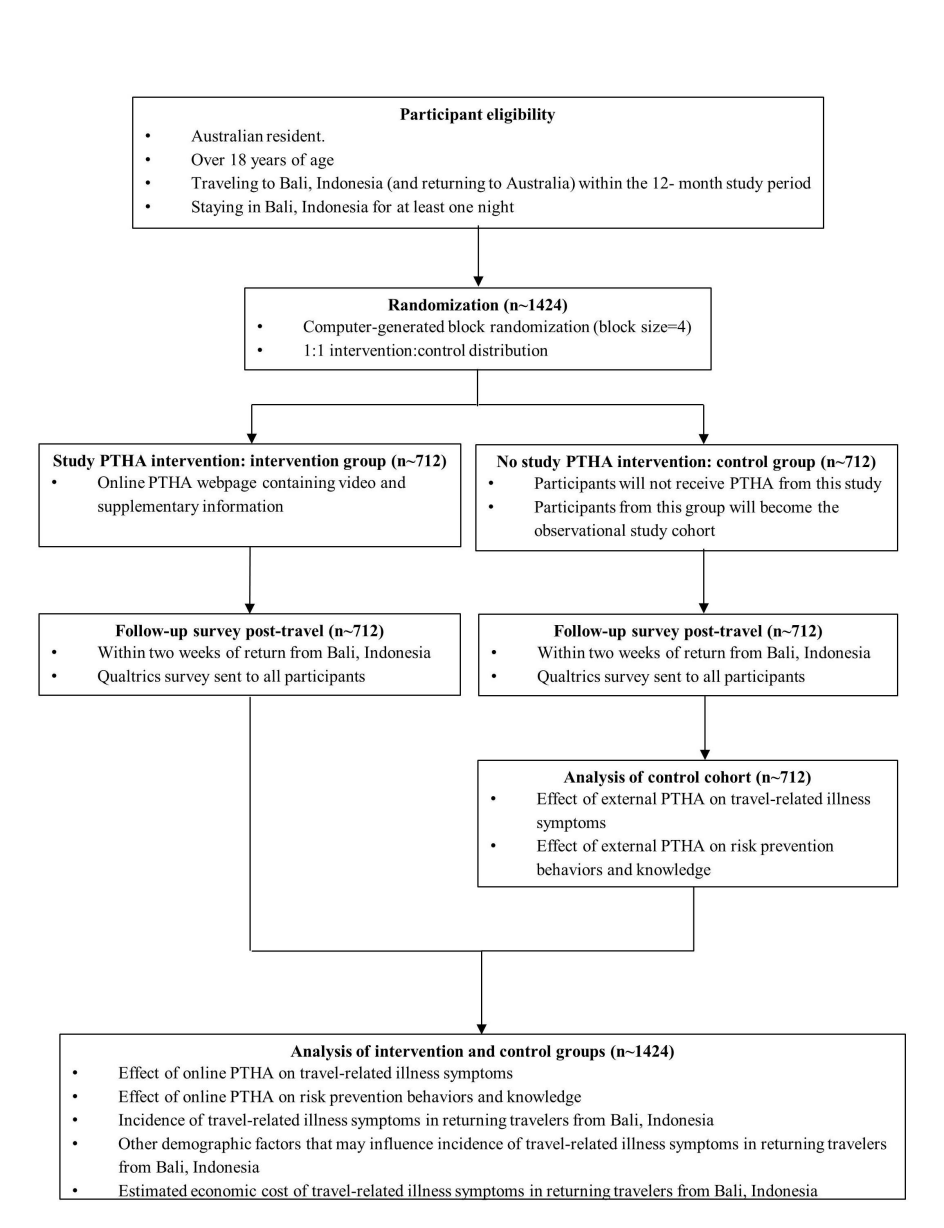
Pretravel health advice (PTHA) among Australians returning from Bali, Indonesia: study design and flow.

To evaluate the impact of PTHA on travel-related illness, rates of self-reported illness in travelers with and without PTHA will be compared (see Figure 1).

#### Study Population

WA residents planning to travel to Bali within a 12-month study period commencing in September 2016 will be eligible for inclusion in this study. Participants must be Australian residents, over the age of 18, traveling to Bali and staying for at least one night, and be willing and able to give consent and agree to complete the survey.

#### Recruitment

Recruitment will take place online, via advertisements promoting the Bali travel survey, strategically located on Facebook and Google pages. The advertisements will run for a 12-month study period to incorporate variation in travel across the year related to seasonal travel or university and school holidays. The advertisements will target people who have searched for Bali flights and/or accommodation online; this information is gathered by Facebook and Google through their single sign-on utility, as well as cookie data to track Internet activity. The main avenue for recruitment is the Internet because a previous study demonstrated that 87% of travelers to Bali make pretravel purchases—flights or accommodation—online [20]. Clicking on the advertisement will take people to a webpage, which briefly describes the study. People interested in enrolling in the study will be asked to enter their departure date, return date, sex, age range, and email address in an online form. This information will allow researchers to determine the age range and sex of those participants who later may be lost to follow-up. Participants will be asked to check a box to indicate that they consent to participate in the study. Travelers can only participate once in the study; multiple enrollments from the same email address will be blocked.

To assist with recruitment, participants will be offered an incentive of entering a lottery to win one AUD $1000 gift voucher or one of 10 AUD $100 gift vouchers. The winning names will be drawn upon study completion. Participants will be informed of the incentive via the online advertisement, consent page, and initial welcome email they receive.

#### Randomization

Randomization will be performed using a custom-built computer algorithm. Participants will be randomly allocated into control or intervention groups after completion of the online enrollment form, and the allocation will be sent to the email management program (MailChimp) used in the study. A block randomization (block size=4) with a uniform 1:1 allocation will be used.

#### Intervention

All participants will receive an email following enrollment containing a welcome message and a reminder to look for an email within two weeks of their return date. For participants allocated to the intervention group, this email will contain the link to the PTHA intervention.

The intervention in this study is a short, approximately 3-minute, online animated video containing PTHA specific to Bali and Southeast Asia. Topics covered include foodborne illness (eg, Bali belly), mosquito-borne illness (eg, dengue, chikungunya, and Zika), avoiding monkey and canine contact (eg, rabies), and checking measles vaccination status. This video is complemented by PTHA in text below the video and the webpage is optimized for mobile devices. This intervention was developed in consultation with a video producer who has previously created videos for the WA Department of Health. The information in the video and text was developed from a review of the travel-related illness literature, as well as reputable websites such as the Australian government smartraveller.org website [21]. This website was the only PTHA source that showed higher levels of health knowledge among travelers to Bali participating in an airport survey in 2014 [20]. The video producers will provide a *video heat map* that graphs each viewing session (ie, where and when the video was watched, which portions were skipped or watched again, and the percentage of the video that each participant watched). The participant may view the video multiple times, accessing it through the email link. The control group will not receive access to this PTHA video, but they may access PTHA on their own accord.

#### Posttravel Follow-Up

Within two weeks of the participants’ nominated return dates, they will receive an email containing a link to the posttravel survey, which will be optimized for mobile devices. The control and intervention groups will receive links to surveys that are almost identical, apart from the wording of one question on PTHA. For the intervention group, this question will be phrased to make it clear they are being asked about PTHA received external to this study (ie, PTHA from their general practitioner or from their own Internet search). If the participant does not complete the survey within a week, a second email will be sent to the participant.

The survey will contain questions in six general themes: (1) information about their trip to Bali, (2) PTHA behaviors, (3) risk prevention behaviors (sample question presented in Textbox 1), (4) knowledge about disease risk (sample question presented in Textbox 2), (5) illness and symptoms during and/or posttravel, and (6) basic demographic information. The survey was adapted from a 2014 WA traveler survey [20] and will take participants, on average, between 5 and 10 minutes to complete, depending on whether or not illness is reported. The final survey and intervention were developed in consultation with WA Department of Health staff and pilot-tested, with the intervention, by a focus group in late 2015.

**Posttravel survey sample questions. Participants will be asked to report the health risk-prevention behaviors they engaged in during travel. Participants may choose from the following responses: Daily, Every 2-3 days, Once a week, and Never.**

Please answer the following questions about your behavior during your recent trip to Bali.

- How often did you use insect repellent on this trip?
- How often did you use alcohol-based hand sanitizer on this trip?
- How often did you use a mosquito net on this trip?
- How often did you use mosquito protective clothing on this trip? (eg, long sleeves)
- How often did you eat eggs with a runny yolk on this trip? (eg, fried egg on top of fried rice)
- How often did you eat fresh fruit you did not peel yourself on this trip?

**Posttravel survey sample questions. Participants will be asked to report their health risk knowledge posttravel. Participants may choose from the following responses: Agree, Disagree, and Unsure.**

The following questions concern your opinions on health risks while traveling.

Please indicate whether you agree or disagree with each of the statements by ticking the appropriate box.

- The risk of someone catching measles in Bali is about the same as catching measles in Australia.
- Mosquitoes that bite during the daytime are a nuisance, but they do not transmit serious diseases.
- The ice from hotels in Bali is safe to serve in drinks.
- It is safe to feed monkeys at tourist venues in Bali.
- Salads (ie, uncooked fruits and vegetables) are safe to eat in Bali.
- You should seek urgent medical attention if you are bitten by a dog or other mammal in Bali.
- It is important to always use insect repellent when outdoors in Bali.
- If you are scratched or bitten by a monkey in Bali, you should receive treatment to prevent rabies.
- You should not eat eggs with runny yolks in Bali.

Participants who report seeking medical attention for a travel-related infection will be asked for permission to obtain specified health information relating to their illness from their health care provider. If consent is given, the participant will be asked to provide some identifying information including name, date of birth, name of medical practice or hospital visited, and the date of medical visit. Participants are advised that the information to be collected from the health care provider will include details regarding relevant diagnosis, laboratory tests, prescriptions, and, if admitted, length of stay. Letters will be sent to health care providers informing them that the participant has consented to participate in the study and that a WA Department of Health employee will contact them within the week by telephone. The WA Department of Health employee will conduct a phone interview with the health care provider using a uniform script. The self-reported data on diagnoses, health services accessed, and treatment will be validated against the health records.

### Study Outcomes and Data Analysis

#### Overview

The primary outcome of the study is a difference in self-reported overall travel-related illness between the intervention and control groups. Secondary outcomes are differences in risk prevention behaviors (Textbox 1) and health risk perceptions (Textbox 2) between the intervention and control groups. The economic cost of travel-related illness symptoms in returning travelers from Bali will also be estimated.

#### Data Analysis: Primary Outcome

Data analysis will be carried out using Epi Info version 7 (Centers for Disease Control and Prevention) and Stata version 12 (StataCorp LP).

The proportion of travelers in the control and intervention groups who self-report illness during their travels, or within two weeks of returning home, will be compared. A univariate analysis will be carried out using Pearson’s chi-square test for comparison of proportions and a *t* test for comparison of mean risk prevention behaviors and knowledge. Logistic regression analyses will be undertaken to estimate the impact of the intervention, while adjusting for other variables such as age, sex, and use of external PTHA, if differences between intervention and control groups are observed at the baseline. Intention-to-treat analysis will be undertaken.

#### Data Analysis: Secondary Outcomes

Data from all participants will also be used to determine the cumulative incidence of illness symptoms in travelers returning from Bali. The impact of demographic factors, risk prevention behaviors, and knowledge on the risk of travel-related illness symptoms in the study population will be analyzed using logistic regression.

Data from the control group will be used to assess further secondary outcomes. A difference in self-reported illness, risk prevention behaviors, and health risk knowledge between those that sourced PTHA independently—external to the study—and those who did not receive any PTHA will be assessed. In addition, self-reported illness symptom rates in travelers returning from Bali will be determined.

The proportion of travelers in the control group that received PTHA from an alternative source, the source of this travel advice, and differences in demographic groups will be compared between travelers who reported illness and those who did not.

#### Cost-of-Illness Analysis

Data from all participants in this study will be used to estimate the cost of travel-related illness in Western Australian travelers to Bali. The cost-of-illness analysis will be carried out using the framework described by Abelson et al [22] to determine the cost of foodborne illness in Australia and New Zealand [23,24]. Costs measured in this analysis will be direct health care costs—including doctor visits, hospitalizations, medical evacuations, and laboratory tests, both in Bali and Australia—and indirect health care costs—including indirect non-health care costs (eg, workdays lost).

#### Sample Size Calculation

The sample size calculation was based upon the online PTHA intervention lowering the rate of self-reported illness in Western Australians returning from Bali by 10%. Existing literature suggests that reduction in illness as a result of visiting a travel doctor before departure is approximately 20% [13,14]. The sample size calculation assumed an illness rate of 40% in the control group [3], with a reduction of 10% in the intervention group, detected at 80% power with a *P* value of .05. A sample size of 712 participants completing the study—356 control and 356 intervention participants—was determined using PS: Power and Sample Size Calculation statistical software [25]. Assuming approximately 50% of participants are lost at follow-up, which is the average for Internet-based trials [26], and although we expect less loss because of the incentive, at least 1424 participants—712 exposed and 712 unexposed participants—would need to be enrolled in the study.

#### Data Management

The Qualtrics survey tool will be used to create and conduct the online survey [27]. All information to and from the Qualtrics server is encrypted using the Transport Layer Security protocol. The Qualtrics servers are protected by Web application firewalls and Qualtrics employs an intrusion detection system to monitor system access for unauthorized uses. Qualtrics data collected in Australia are stored in Sydney, Australia.

### Strengths and Limitations

There are multiple benefits to using our proposed study design for this project. It will be possible to simultaneously determine base rates of illness and compare existing PTHA practices (ie, PTHA sourced by the participants independently) and a novel intervention.

Limitations include the potential exclusion of those travelers without access to, or unfamiliarity with, a computer or mobile phone; however, 87% of WA travelers to Bali used the Internet to purchase flights and/or accommodation [20]. Also, 2015 social media statistics report that 82% of Australian Internet users use Google and 60% use Facebook each month for around three and eight hours, respectively [28]. In addition, the PTHA intervention is targeted to, and intended for use by, Internet-savvy consumers.

It is possible that participants with an interest in health will be more likely to enroll in this study. This may lead to an over-reporting of PTHA-seeking and risk-prevention behaviors. To minimize this effect, the online advertising and study enrollment webpage will have an emphasis on the Bali travel experience more generally and will not highlight the health risks associated with travel to this destination. In addition, the results from this study will be compared to the results from the 2014 airport survey previously undertaken by our group, which used different methodology. The demographics of the two study cohorts will be compared to determine how the online cohort compares to the cohort recruited at the airport. Participants may also claim more “favorable” health-seeking behaviors when providing health information to a health authority. To reduce this effect, the surveys have been designed to be self-administered.

The cost-of-illness analysis has several limitations. While every effort will be made to follow up with participants who report visiting a health care professional about their illness, this may not always be possible. The participant may decline to provide their information, this information may be incorrect, and some health care professionals may decline to provide the required information. In addition, if the participant has reported illness with ongoing treatment, it may be difficult to determine an accurate cost.

### Ethics

Ethical approval has been obtained from the Western Australian Department of Health Human Research Ethics Committee (RA/4/1/8110) and the University of Western Australia Health Human Research Ethics Committee (Project #2015/58). This study has been registered with the ANZCTR (trial ID: ACTRN12615001230549).

## Results

The study began recruitment in September 2016 and will conclude in September 2017. Data analysis will take place in late 2017, with results disseminated via peer-reviewed journals in early 2018.

## Discussion

Approximately 10-20% of Western Australians visit Bali each year [1] and are exposed to the increased risk of illness associated with traveling to a developing nation. There are limited data regarding the travel-related illness burden, as infections may be non-notifiable, or notifiable but underdiagnosed and under-reported. Still, existing notifiable disease data suggest Bali travel contributes a significant burden of disease to Western Australia. Internet-based preventative measures present an opportunity for health departments to learn how to better protect the health of their residents abroad. As the number of notifiable disease cases attributed to Indonesia has risen in Western Australia [4], it is clear that current strategies are not adequate.

There have been very few studies, and no randomized controlled trials, determining the impact of PTHA on travel-related illnesses [13,14,19]. It is hoped that the results of this study may have broad applications locally and internationally. If a strategy of delivering PTHA online can prove effective, this intervention could be implemented more broadly.

## Abbreviations

PTHA: pretravel health advice
WA: Western Australian
